# IL-33 Protects Mice against DSS-Induced Chronic Colitis by Increasing Both Regulatory B Cell and Regulatory T Cell Responses as Well as Decreasing Th17 Cell Response

**DOI:** 10.1155/2018/1827901

**Published:** 2018-11-13

**Authors:** Jun-feng Zhu, Ying Xu, Jian Zhao, Xue Li, Xinrui Meng, Tian-qi Wang, Ben-yao Zou, Peng-yan Zhao, Qi Liu, Chang-long Lu, Fang-liang Zheng, Hong-sheng Liu

**Affiliations:** ^1^Life Science School, Liaoning University, Shenyang 110036, China; ^2^Engineering Laboratory for Molecular Simulation and Designing of Drug Molecules of Liaoning, Shenyang 110036, China; ^3^Department of Immunology, China Medical University, Shenyang 110013, China; ^4^Research Center for Computer Simulating and Information Processing of Bio-Macromolecules of Liaoning, Shenyang 110036, China

## Abstract

**Background:**

Previously, we have reported that IL-33 functioned as a protective modulator in dextran sulfate sodium- (DSS-) induced chronic colitis by suppressing Th17 cell response in colon lamina propria and IL-33 induced both regulatory B cells (Bregs) and regulatory T cells (Tregs) in mesenteric lymph nodes (MLNs) of mice with DSS-induced acute colitis. Moreover, we speculated that IL-33 would promote the Treg or Breg responses leading to the attenuation of DSS-induced chronic colitis. So, we investigated the role of IL-33 on Bregs and Tregs in the MLN of DSS-induced chronic colitis mice.

**Methods:**

IL-33 was administered by intraperitoneal injection to mice with DSS-induced chronic colitis. Clinical symptoms, colon length, and histological changes were determined. The production of cytokines was measured by ELISA. The T and B cell subsets were measured by flow cytometry. The expression of mRNA of transcription factors was measured by quantitative real-time PCR.

**Results:**

We show that IL-33 treatment increases both Breg and Treg responses in the MLN of mice with DSS-induced chronic colitis. Moreover, IL-33 treatment also decreases Th17 cell response in the MLN of mice with DSS-induced chronic colitis.

**Conclusion:**

Our data provide clear evidence that IL-33 plays a protective role in DSS-induced chronic colitis, which is closely related to increasing Breg and Treg responses in the MLN of mice as well as suppressing Th17 cell responses.

## 1. Introduction

Inflammatory bowel disease (IBD), including ulcerative colitis (UC) and Crohn's disease (CD), is a chronic inflammatory condition provoked by an aberrant innate and/or adaptive immunity against the bacterial flora in a genetically predisposed host [1]. The available evidence suggests that CD is characterized by a Th1/Th17 response [2–5], while UC is associated with the overproduction of Th2-type cytokines such as IL-5 and IL-13 [6–8]. Dextran sulfate sodium- (DSS-) induced chronic colitis is characterized by a predominant Th17/Th1-mediated immune response and mucosal inflammation which closely resembles important immunological aspects of CD [9–11].

IL-33, also known as interleukin-1 family member 11 (IL-1F11), was identified as a novel member of the IL-1 family. IL-33 is synthesized as a 30 kDa precursor protein and can be cleaved by caspase-1 to become an 18 kDa mature protein [12]. IL-33 is expressed in macrophages, dendritic cells, fibroblasts, endothelial cells, and intestinal epithelial cells [13–15]. IL-33 signals via a heteromeric receptor that consists of ST2L (or ST2) and IL-1R accessory protein (IL-1RAcP) [16]. ST2 is mainly expressed on activated Th2 cells and Tregs [17].

Multiple studies have already demonstrated that IL-33 was induced in the intestinal mucosa of patients with IBD and an IL-33 polymorphism has been associated with IBD [18–21]. However, the main role of IL-33 in IBD is complicated and remains to be elucidated. In the Th2-mediated UC and its animal models, IL-33 plays a pathogenic role associated with type 2 immune responses [22–24]. However, by switching Th17/Th1 to Th2-type immune response, IL-33 can reduce the development of CD and its animal models, which are mainly mediated by Th17 and Th1 response [25–27]. The above observations suggest that IL-33 is involved in the pathogenesis of IBD.

We formerly showed that IL-33 alleviated DSS-induced chronic colitis by suppressing Th17 cell response in colon lamina propria [28]. Moreover, our previous data have shown that IL-33 treatment led to a marked deterioration in both the clinical and histopathological aspects of the DSS-induced acute colitis by enhancing Th2 cell responses but increasing both regulatory T cell and regulatory B cell responses in the mesenteric lymph nodes (MLNs) [29]. Despite these advances, it is not yet known whether IL-33 played a role in the MLN during the development of DSS-induced chronic colitis. And we speculate that IL-33 would promote the Treg or Breg responses leading to the attenuation of DSS-induced chronic colitis. Furthermore, DSS-induced acute colitis was known as a UC model, and the DSS-induced chronic colitis have long been considered as a Th1-type colitis animal model resembling CD; the MLNs act as an effector tissue in gastrointestinal inflammation [30], so we sought to elucidate the role of IL-33 in the MLN during the development of DSS-induced chronic colitis.

## 2. Materials and Methods

### 2.1. Animals

Specific pathogen-free male C57BL/6 mice aged 7 weeks and weighing 20–22 g were purchased from Beijing HFK Bioscience Co. Ltd. (Beijing, China). The mice were maintained and bred under specific pathogen-free conditions (temperature 24–25°C, humidity 70%–75%, and a 12 h light/12 h dark lighting regimen). The study protocol was approved by the China Medical University Animal Care and Use Committee.

### 2.2. Production of Recombinant Mouse IL-33 Protein and Assessment of Its Activity

Expression and purification of rIL-33, removal of the endotoxin, and assessment of biological activity of rIL-33 were carried out as previously described [28].

### 2.3. Induction and Evaluation of Chronic Colitis Induced by DSS

Induction of chronic colitis and administration of rIL-33 were carried out as previously described [28]. The severity of colitis was assessed using the disease activity index (DAI) based on weight loss, occult blood in stool, and hematochezia; the scores are described in Table 1. After sacrifice on day 30, the colons were removed and their lengths were measured. Meanwhile, MLN cells were extracted for analysis by flow cytometry, quantitative real-time PCR, and ELISA.

### 2.4. Histological Assessment of Colitis

The colon specimens were fixed with 4% paraformaldehyde and embedded in paraffin, and sections 4 *μ*m thick were stained with hematoxylin and eosin to evaluate colonic histology. The degree of colitis was assessed as previously described [28].

### 2.5. MLN Cell Isolation and Stimulation

MLNs from individual mice were collected under sterile conditions in ice-cold PBS with 10% fetal calf serum (FBS). Then, the lymph nodes were gently disrupted with a sterile syringe plunger and filtered through a nylon cell strainer (40 *μ*m mesh; BD Biosciences, San Jose, CA, USA). The cells were collected after centrifugation at 1500 rpm at room temperature for 5 min.

MLN cells (5 × 10^5^/well) were cultured in 96-well plates (Falcon; BD Biosciences) with 0.2 mL RPMI1640 (supplemented with 10% FBS and 1% penicillin/streptomycin) and stimulated with anti-CD3 (10 *μ*g/mL eBioscience, San Diego, CA, United States) and anti-CD28 (1 *μ*g/mL, eBioscience) mAb for 48 h.

### 2.6. Cytokine Analysis by Enzyme-Linked Immunosorbent Assay (ELISA)

The cytokine concentrations were measured using mouse immunoassay kits (R&D Systems) according to the manufacturer's instruction. The levels of IL-6, IL-23, IL-1*β*, IL-12p70, and TNF-*α* were measured in supernatants without anti-CD3/antiCD28 mAb stimulations. The levels of IFN-*γ*, IL-17A, IL-10, IL-4, IL-5, and IL-13 were measured in supernatants with or without anti-CD28/anti-CD3 mAb stimulations.

### 2.7. Confirmation of mRNA Expression of the Transcription Factor by Quantitative Real-Time PCR

The quantitative real-time PCR was performed according to the methods described previously [28]. Briefly, total RNA was extracted from cells isolated from the MLN using the RNAiso Plus (Takara, Dalian, China). The first-strand cDNA was generated by using PrimeScript™ RT Reagent Kit with gDNA Eraser (Perfect Real Time, Takara). The PCR mixture was prepared by using SYBR® Premix Ex Taq™ (Tli RNaseH Plus, Takara) and one of the primers listed in Table 2.

### 2.8. Flow Cytometry Analysis

Flow cytometry analysis was performed according to the methods described previously [28]. Briefly, the cells isolated from the MLN (the MLN cells) were incubated with an Fc*γ* receptor-blocking mAb (CD16/32; 2.4G2, BD Biosciences) for 15 minutes at 4°C. For surface antigen detection, the cells were labeled with monoclonal antibodies against Gr-1 (RB6-8C5, BD Biosciences), F4/80 (BM8, BD Biosciences), *αβ*TCR (H57-597, BD BioLegend), *γδ*TCR (GL3, BioLegend), NK1.1 (PK136, BioLegend), CD4 (RM4-5, BD Biosciences), CD44 (IM7, BD Biosciences), CD25 (PC61, BioLegend), B220 (RA3-6B2, BioLegend), and CD19 (MB19-1, BioLegend) for 30 min at 4°C.

For intracellular cytokine staining, the cells isolated from the MLN (the MLN cells) were stimulated with ionomycin (1 mg/mL; Sigma-Aldrich) and PMA (25 ng/mL; Sigma-Aldrich) for 5 h at 37°C, with brefeldin A (10 mg/mL; Sigma-Aldrich) added after 1 h. Then, the cells were fixed and permeabilized with fixation/permeabilization working solution for 20 min at 4°C followed by incubation with monoclonal antibodies against IFN-*γ* (XMG1.2, BD Biosciences), IL-17A (TC11-18H10.1, BD Biosciences), and IL-10 (JES5-16E3, BD Biosciences). The cells were analyzed using a Cytofix/Cytoperm Plus Kit.

### 2.9. Statistical Analysis

Statistical significance was calculated by Student's *t*-test using Prism software (GraphPad Prism Software, La Jolla, CA). Differences with *p* values of 0.05 were considered statistically significant.

## 3. Results

### 3.1. IL-33 Treatment Attenuates DSS-Induced Chronic Intestinal Inflammation in Mice

**Figure 1:**
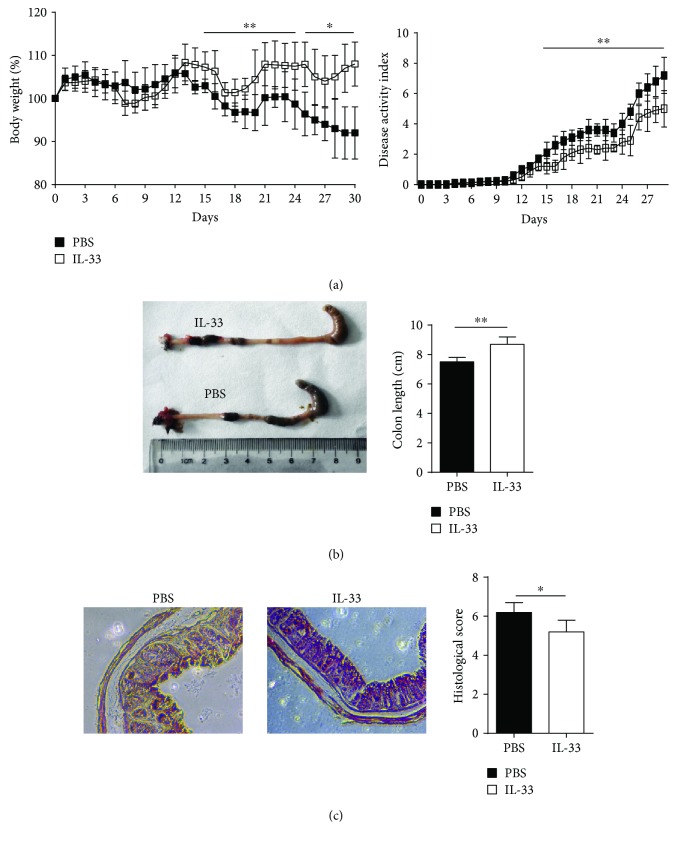
IL-33 treatment attenuates DSS-induced chronic colitis in the C57BL/6 mice. Mice were orally treated with 2% DSS in the drinking water to induce colitis as described in Materials and Methods. (a) Body weight and disease activity index were daily observed. On day 30, (b) macroscopic changes, colon length, and (c) histological score (original magnification, ×200) were analyzed. Data indicate mean ± SD of each group (*n* = 8–10/group) obtained from a representative of three independent experiments (^∗^*p* < 0.05, ^∗∗^*p* < 0.01).

To directly assess the role of IL-33 in colitis development, we administered recombinant IL-33 i.p. to DSS-administered mice as described in Materials and Methods. As shown in Figure 1(a), IL-33-treated mice displayed alleviative intestinal inflammation as indicated by the attenuation of body weight loss and obviously decreased DAI score compared with PBS-treated mice. On macroscopic examinations, the colon of PBS-treated mice was markedly shorter than that of IL-33-treated mice on day 30 (Figure 1(b)). Meanwhile, histological examination showed that crypt damage, ulceration, and infiltration of inflammatory cells were markedly aggravated in the colons of PBS-treated mice as compared with IL-33-treated mice. The histological score of the colons was remarkably higher in PBS-treated mice than that in IL-33-treated mice (Figure 1(c)). These data indicated a crucial beneficial effect of IL-33 on DSS-induced chronic colitis in mice.

### 3.2. IL-33 Alters the Accumulation of Immune Cells in the MLN

**Figure 2:**
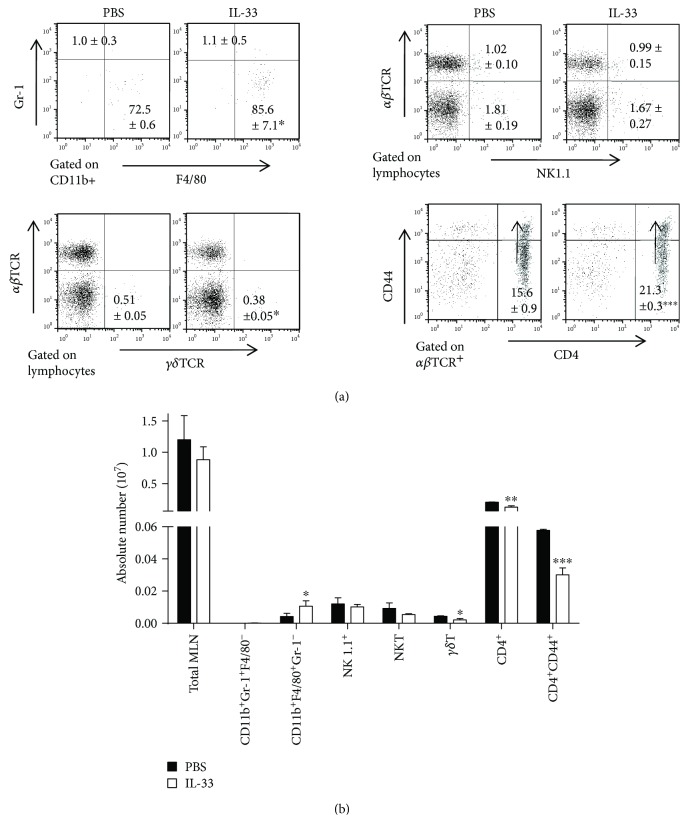
Lymphocyte subsets of the MLN of IL-33- and PBS-treated mice with DSS-induced chronic colitis were analyzed by flow cytometry. (a) The frequency of neutrophil (CD11b^+^Gr1^+^F4/80^−^), macrophage (CD11b^+^Gr1^−^F4/80^+^), CD4^+^ T cell, CD4^+^CD44^+^ T cells (effector T cells), *γδ*T cell (*γδ*TCR), NK cells (NK1.1^+^), or NKT cell (NK1.1^+^*αβ*TCR^+^) in cells isolated from the MLN on day 30 after administration of DSS. (b) The absolute number of above cells on the same day. Data indicate mean ± SD of each group (*n* = 5/group) obtained from a representative of three independent experiments. Statistically significant differences are shown (^∗^*p* < 0.05, ^∗∗^*p* < 0.01, or ^∗∗∗^*p* < 0.001).

No remarkable differences in the percentages and absolute numbers of neutrophils (CD11b^+^F4/80^−^Gr-1^+^) and NK cells (NK1.1^+^*αβ*TCR^−^) in the MLN of IL-33-treated mice and PBS-treated mice were observed. As shown in Figure 2, the percentage and the absolute numbers of macrophage (CD11b^+^Gr-1^−^F4/80^+^) in the MLN were dramatically higher in the IL-33-treated chronic colitis group than in the control group. Compared to the control group, the absolute number of NKT (NK1.1^+^*αβ*TCR^+^) and *γδ*T cells in the MLN significantly decreased in the IL-33-treated chronic colitis group, while the percentage of NKT (NK1.1^+^*αβ*TCR^+^) and *γδ*T cells showed no differences in the two groups. We also examined the populations of T cell subsets in the MLN. The percentage of CD4^+^CD44^+^ cells in CD4^+^ T-MLN cells of mice treated with IL-33 was higher than that in the control mice, but the absolute numbers of CD4^+^ and CD4^+^CD44^+^ T-MLN cells of IL-33-treated mice reduced as compared to the control mice.

### 3.3. IL-33 Alters the Cytokine Profile in the MLN

**Figure 3:**
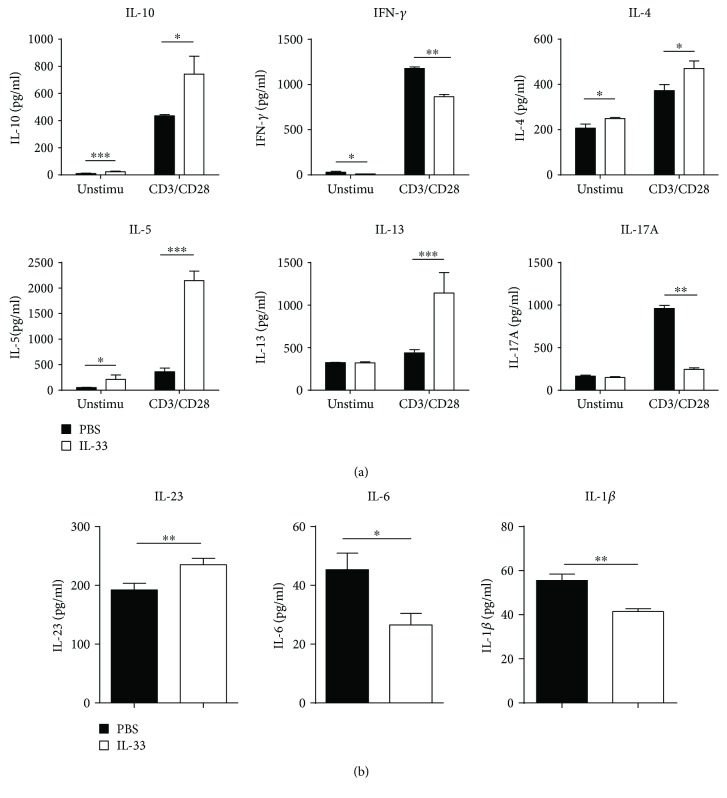
Cytokine production by lymphocytes isolated from the MLN of IL-33- and PBS-treated mice with DSS-induced colitis. (a) MLN cells with or without anti-CD3 and anti-CD28 mAb (CD3/CD28) stimulations. (b) MLN cells without any stimulation. Data indicate mean ± SD of each group (*n* = 5/group) obtained from a representative of three independent experiments and were valued by a Student *t*-test (^∗^*p* < 0.05, ^∗∗^*p* < 0.01, or ^∗∗∗^*p* < 0.001).

As shown in Figure 3, measurement of cytokine concentrations in the culture supernatants of the MLN without any stimulation manifested that the levels of IL-6, IL-1*β*, and IL-23 were sharply decreased in the IL-33-treated chronic colitis group compared with the control group. In addition, low levels of TNF-*α* and IL-12p70 were detected, although there were no indispensable differences between the two groups (data not shown). The level of IFN-*γ* with anti-CD3 and anti-CD28 mAb stimulation and without any stimulation was strikingly reduced in the IL-33-treated chronic colitis group compared with the control group. The level of IL-17A with anti-CD3 and anti-CD28 mAb stimulation was noticeably reduced in the IL-33-treated chronic colitis group compared with the control group, while a similar concentration of IL-17A without any stimulation was observed in these two groups (Figure 3). The level of IL-4 and IL-5 with anti-CD3 and anti-CD28 mAb stimulation and without any stimulation was markedly increased in the IL-33-treated chronic colitis group compared with the control group (Figure 3). The level of IL-13 was noticeably increased with anti-CD3 and anti-CD28 mAb stimulation, and similar results were observed without any stimulation in the supernatants of the MLN of the IL-33-treated chronic colitis group compared with the control group (Figure 3).

### 3.4. IL-33 Shifts Th Cell-Mediated Immune Responses in the MLN of Mice with DSS-Induced Chronic Colitis

**Figure 4:**
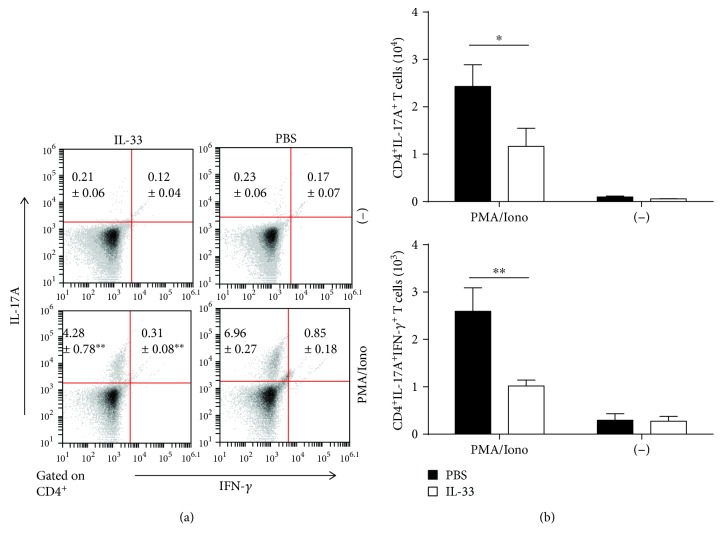
The population of cytokine-producing T cells in the MLN of IL-33- and PBS-treated mice with DSS-induced colitis was analyzed by flow cytometry. (a) The frequencies and the absolute numbers of CD4^+^IL-17A^+^ and CD4^+^IL-17A^+^IFN-*γ*^+^ T cells in the MLN of IL-33- and PBS-treated mice with DSS-induced chronic colitis. (b) The absolute numbers of cytokine-producing CD4^+^IL-17A^+^ and CD4^+^IL-17A^+^IFN-*γ*^+^ T cells of the MLN with and without stimulation. Data indicate mean ± SD of each group (*n* = 3/group) obtained from a representative of three independent experiments. Statistically significant differences are shown (^∗^*p* < 0.05, ^∗∗^*p* < 0.01).

Furthermore, we detected IFN-*γ* or IL-17A-producing CD4^+^ T cells in the MLN of IL-33-treated and untreated chronic colitis mice. There was a critical decrease in the percentage and absolute number of IL-17A-producing CD4^+^ T cells in the MLN of IL-33-treated mice compared with model mice after PMA/ionomycin stimulations. The percentages and absolute numbers of IFN-*γ*-producing CD4^+^ T cells in the MLN showed no marked differences between these two groups (Figure 4). Moreover, there was a marked reduction in the frequency of IL-17A^+^IFN-*γ*^+^ double-producing CD4^+^ T cells in the MLN of IL-33-treated mice, compared to PBS-treated mice, with a marked reduction in the absolute number of these cells (Figure 4). Moreover, compared to the chronic colitis mice treated with PBS, those mice treated with IL-33 showed an enormous reduction in the expression of ROR-*γ*t mRNAs. These results suggested that the effect of IL-33 may suppress Th17 cell responses in the MLN of DSS-induced chronic colitis mice.

### 3.5. IL-33 Increases Both Regulatory T Cell (Treg) and Regulatory B Cell (Breg) Responses in the MLN during DSS-Induced Chronic Colitis

**Figure 5:**
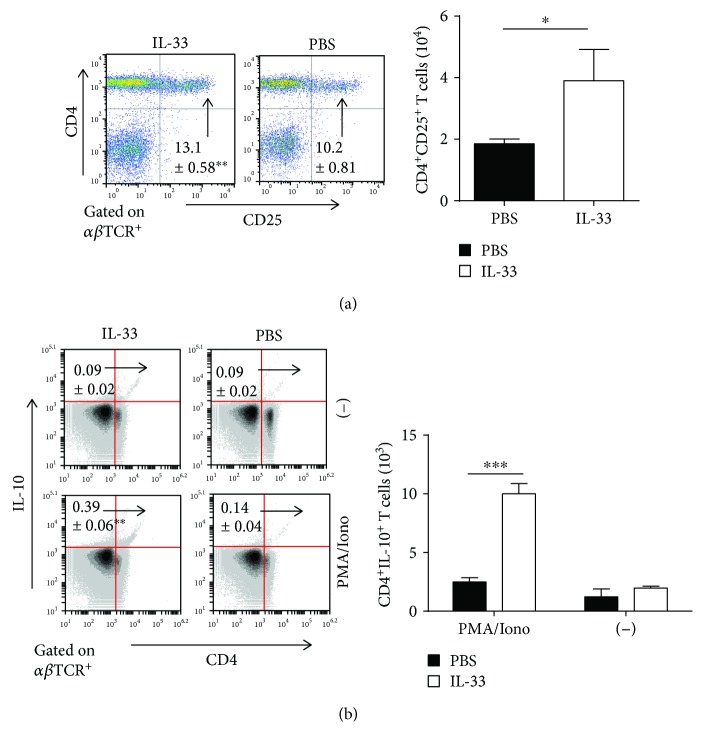
IL-33 increased Treg responses in the MLN of mice with DSS-induced chronic colitis. (a) The frequencies and the absolute numbers of CD4^+^CD25^+^ T cell in the MLN. (b) The percentage and the absolute numbers of cytokine-producing CD4^+^IL-10^+^ T cells in lymphocytes of the MLN with and without stimulation. Data indicate mean ± SD of each group (*n* = 3/group) obtained from a representative of three independent experiments and were valued by a Student *t*-test. (^∗^*p* < 0.05, ^∗∗^*p* < 0.01, or ^∗∗∗^*p* < 0.001).

**Figure 6:**
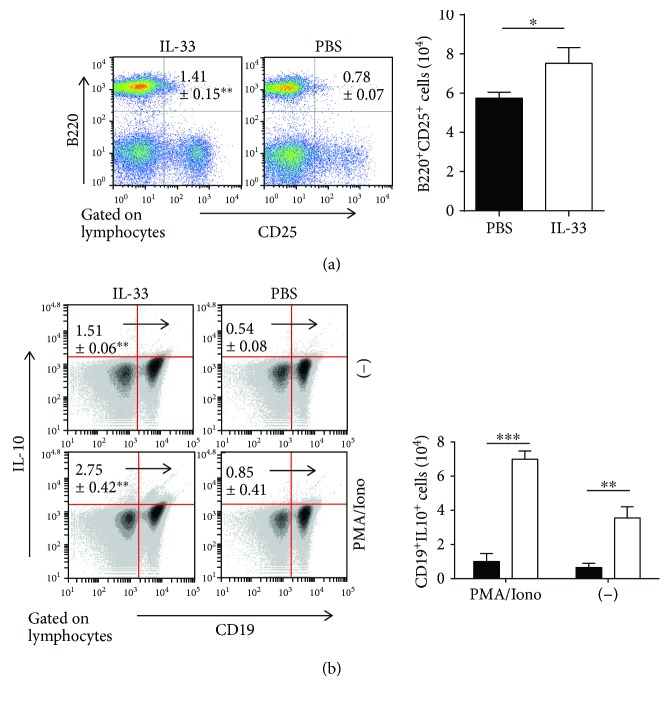
IL-33 promoted Breg responses in the MLN of mice with DSS-induced chronic colitis. (a) The frequencies and the absolute numbers of B220^+^CD25^+^ cell in the MLN. (b) The percentage and the absolute numbers of cytokine-producing CD19^+^IL-10^+^ cells in the MLN with and without stimulation. Data indicate mean ± SD of each group (*n* = 3/group) obtained from a representative of three independent experiments and were valued by a Student *t*-test. (^∗^*p* < 0.05, ^∗∗^*p* < 0.01, or ^∗∗∗^*p* < 0.001).

**Figure 7:**
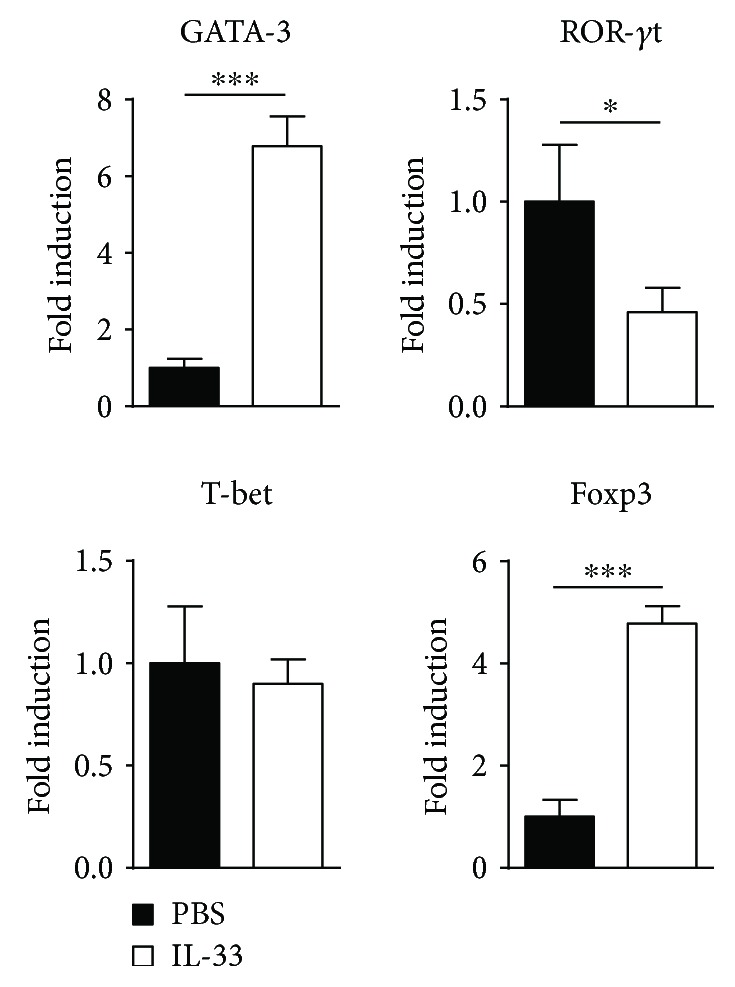
mRNA expression changes of the transcription factor in the MLN. Data indicate mean ± SD of each group (*n* = 6/group) obtained from a representative of three independent experiments and were valued by a Student *t*-test (^∗^*p* < 0.05, ^∗∗∗^*p* < 0.001).

Tregs and Bregs could protect mice from DSS-induced intestinal inflammation [31, 32]. Next, we investigated the effect of IL-33 on both Treg and Breg responses in the MLN of mice with DSS-induced chronic colitis. The percentages and absolute numbers of CD25-expressing T cells (CD4^+^CD25^+^) and CD25-expressing B cells (B220^+^CD25^+^) in T-LPLs were drastically higher in the MLN of the IL-33-treated chronic colitis group than that of the control group (Figures 5(a) and 6(a)). Furthermore, there was a substantial increase in the percentage and absolute number of CD4^+^IL-10^+^ T cells and CD19^+^IL-10^+^ cells in IL-33-treated chronic mice compared with control mice (Figures 5(b) and 6(b)). The level of IL-10 with anti-CD3 and anti-CD28 mAb stimulation and without any stimulation was significantly increased in the IL-33-treated chronic colitis group compared with the control group (Figure 3(a)). Moreover, compared to the chronic colitis mice treated with PBS, those mice treated with IL-33 showed a marked increase in the expression of Foxp3 mRNAs (Figure 7). These results provide evidence that IL-33 plays an important role in upregulating both Treg and Breg responses in the MLN in our experimental system.

## 4. Discussion

Several years ago, UC was designated as a Th2-mediated disease based on the elevated production of IL-5 and IL-13, whereas T cells in CD produce excessive amounts of Th1-type cytokines, such as IFN-*γ* and IL-12 [33–35]. However, the Th1/Th2 paradigm was modified with the discovery of Th17 [36]. The DSS-induced colitis model is widely used because of its simplicity and many phenotypical similarities with human IBD [37]. DSS-induced acute colitis is characterized by diarrhea, bloody faeces, weight loss, and a histological picture of inflammation and ulceration as seen in UC [38]. Moreover, the adaptive immune responses mediated by Th2 cells play a needful role in the pathogenesis of DSS-induced acute colitis [39]. However, the high Th1 cytokines (IL-12, IFN-*γ*, IL-1, and TNF-*α*) and Th17 cytokine (IL-17) productions were observed in colon of the DSS-induced chronic colitis model. A different cytokine profile in the chronic phase of DSS colitis was found between acute and chronic phases of DSS-induced colitis, which reflected that DSS-induced acute colitis should be known as a UC model and the DSS-induced chronic colitis should be considered as a Th1/Th17-type colitis animal model resembling CD.

The Th17 pathway was shown to be very important in the pathogenesis of human IBD [40]. Moreover, recent studies have shown that Th17 cells are also responsible for the development of DSS-induced chronic colitis [9, 11]. There are several studies reported that IL-33 suppressed Th17 response in autoimmune disease [41–43]. Our results also showed that the percentage and the absolute number of Th17 cells in the MLN were markedly lower in IL-33-treated mice than in PBS-treated mice. Moreover, we observed that the level of ROR-*γ*t, which directed Th17 differentiation [44], was lower in the MLN of IL-33-treated mice than PBS-treated mice. IL-23 promoted intestinal Th17 cell accumulation and enhanced the emergence of an IL-17A^+^IFN-*γ*^+^ population of T cells through direct signaling into T cells [45]. Accordingly, IL-33 treatment decreased IL-23 production in the MLN of DSS-treated mice, which suggested that IL-33 suppressed Th17 response depending on IL-23 signaling. However, we did not find that IL-33 suppressed the Th17 response in the MLN of mice with DSS-induced acute colitis [29]. The most plausible explanation for our observation is that the level of Th17 response in the acute phase of DSS-induced colitis is very low but high in the chronic phase.

Tregs are known to play a key role in the pathogenesis of IBD, as well as in other autoimmune disorders [46–49]. Tregs regulate immune responses that are dependent on the expression of the IL-10 and the transcription factor Foxp3 [50]. IL-10 may play an important role in the suppressive function of Tregs [51]. A recent study has shown that IL-33 can induce Tregs by stimulating IL-2 secretion by CD11c^+^ DC both in vitro and in vivo [52]. Duan et al. reported that IL-33 treatment protected mice against TNBS-induced colitis by promoting Foxp3^+^ Treg response [27]. Similarly, we found that IL-33 can induce CD4^+^IL-10^+^ Tregs in the MLN of mice with DSS-induced acute colitis [29]. In accordance with above observation, our data demonstrated that the expression level of IL-10, the absolute number and percentage of CD4^+^CD25^+^ and CD4^+^IL-10^+^ Tregs in MLN of mice with DSS-induced chronic colitis, were strikingly higher in the IL-33 group than in PBS group.

Over the years, a novel subset of B cells named Bregs, which exert unique immune regulatory functions to modulate inflammation and autoimmunity, has recently emerged [53, 54]. Similar to Tregs, Bregs exert regulatory functions via the production of cytokines, such as IL-10 and TGF-*β*, and express inhibitory molecules that suppress pathogenic T cells and induce Tregs [53, 55]. It is reported that MLN B cells protect mice from colitis induced by CD4^+^CD45RB^hi^ T cells [56]. And a B cell subset appears under a chronic inflammatory environment, produces IL-10, and suppresses the progression of intestinal inflammation [57]. It is reported that Breg dysfunction induced intestinal inflammation in SAMP1/Yit (SAMP1) murine recognized as a model of human CD [58, 59]. And IL-10 production from regulatory B10 cells regulates DSS-induced intestinal injury [60]. These findings showed that IL-10-producing MLN Bregs, induced by the chronic intestinal inflammatory environment, could suppress the enteric inflammation. Mizoguchi et al. [57] reported that Breg induced by IL-33 could be responsible in protecting the WT mice from colitis and adoptive transferring the Breg^(IL-33)^ into IL-10KO mice could also block the development of spontaneous IBD. Likewise, our previous study showed that IL-33 can induce Bregs in mice with DSS-induced acute colitis [29]. Consistent with this, in this study, we found that IL-33 induces Bregs (CD19^+^CD25^+^) and IL-10-producing Bregs (CD19^+^IL-10^+^) in the MLN of mice with DSS-induced chronic colitis. In addition, we found the level of Breg responses promoted by IL-33 was higher in the chronic phase of colitis than the acute phase. Chronic intestinal inflammatory condition generates an IL-10-producing regulatory B cell subset [57]. So, the level of Breg responses promoted by IL-33 was higher in the chronic phase of colitis than the acute phase. The higher level of Breg responses may be one of the reasons why IL-33 can suppress the DSS-induced chronic colitis.

*γδ*T cells, which are a minor subset of T lymphocytes, play a protective role in acute DSS colitis but are involved in the exacerbation of chronic colitis [61, 62], suggesting that *γδ*T cells may be a promising target in the treatment of IBD. Treg can inhibit the production of IFN-*γ* by antigen-specific memory *γδ*T cells [63]. In the current study, the absolute numbers and percentages of *γδ*T cells in the MLN were dramatically lower in the IL-33 group than in the PBS group. That may be another reason for the downregulation expression of IFN-*γ*.

Macrophages are divided into M1 and M2 types in response to the different stimuli in the local microenvironment [64]. Macrophages develop into the proinflammatory M1 phenotype in response to IFN-*γ*, while the anti-inflammatory M2 type is induced by type 2 cytokines including IL-4 and IL-13 [65]. M1 macrophages contribute to the pathogenesis of DSS-induced colitis primarily by secreting proinflammatory cytokines and causing tissue damage. In contrast, M2 macrophages contribute to the resolution of DSS-induced colitis primarily by expressing low levels of proinflammatory cytokines [66]. The recent study showed that IL-33-induced M2-type macrophage attenuates the development of TNBS-induced colitis [67]. In the present study, IL-1*β* and IL-6, which were mainly produced by M1-type macrophage, decreased in the MLN of IL-33-treated mice but the production of spontaneous IL-10 by M2-type macrophage increased; moreover, the percentage and absolute number of macrophage in the MLN of the IL-33 group were distinctly higher than that of PBS the group. So, the macrophages induced by IL-33 in our experimental system may be M2 type.

Due to the high complexity of human IBD, it is hard to define IL-33 as a therapeutic target. In fact, IL-33 appears to enhance intestinal inflammation in the acute phase of DSS colitis and possibly in UC patients which are driven by Th2 type and innate immune responses. Conversely, IL-33's effects in a Th1-driven model, such as in TNBS colitis or DSS-induced chronic colitis, may result in decreased intestinal inflammation mediated by cytokine and cell-mediated modulation of immune responses. IL-33 might be helpful to counterbalance excessive Th1-based immune responses, such as CD, but not UC which is driven by Th2-type immune responses.

## 5. Conclusion

In conclusion, our data here shows that IL-33 treatment substantially ameliorates the development of DSS-induced chronic colitis by decreasing Th17 cell response and increasing both regulatory B cell and regulatory T cell responses. We represent strong evidence that IL-33 might offer an alternative therapeutic method for managing CD.

## Figures and Tables

**Table 1:** Disease activity index score chart.

Body weight loss	Stool consistency	Stool blood	Integral
0	Normal	Normal	0
1%–5%			1
5%–10%	Loose stool	Fecal occult blood test positive	2
10%–20%			3
>20%	Diarrhea	Gross bleeding	4

**Table 2:** Primer sequences for qPCR.

Gene	Forward	Reverse
Foxp3	GGCCCTTCTCCAGGACAGA	GCTGATCATGGCTGGGTTGT
GATA-3	ACAGCTCTGGACTCTTCCCA	GTTCACACACTCCCTGCCTT
T-bet	CCAGGGAACCGCTTATATGT	CTGGGTCACATTGTTGGAAG
ROR-*γ*t	CCACTGCATTCCCAGTTTCT	CGTAGAAGGTCCTCCAGTCG
*β*-Actin	TTCCAGCGTTCCTTCTTGGGT	GTTGGCATAGAGGTGTTTACG

## Data Availability

The data that support the findings of this study are available from the corresponding author Kristien Van Belle upon reasonable request.
